# Nurses' experiences of recruitment and migration from developing countries: a phenomenological approach

**DOI:** 10.1186/1478-4491-5-15

**Published:** 2007-06-07

**Authors:** Paul H Troy, Laura A Wyness, Eilish McAuliffe

**Affiliations:** 1 Beaumont Hospital, P.O Box 1297, Beaumont Road, Dublin 9, Ireland; 2 Health Policy and Management, Trinity College Dublin, 3-4 Foster Place, Dublin 2, Ireland; 3 Centre for Global Health, Trinity College Dublin, 3-4 Foster Place, Dublin 2, Ireland

## Abstract

**Background:**

There is growing concern globally at the current flows of nurse migration, particularly from low-income to middle and high-income countries. Recruitment practices of many countries such as Ireland are thought to be fuelling this rate of migration. This paper aims to establish the perceptions and opinions of those involved in the recruitment process on their role in recruitment and the effects recruitment has on both source and destination countries.

**Methods:**

A purposive sample of 12 directors of nursing, from major academic teaching hospitals in Dublin and hospitals in South Africa and the Philippines were recruited. Ten overseas nurses were also recruited. A phenomenological approach was used with semi-structured interviews as the data collection method.

**Results:**

There were pronounced differences in opinions between the Irish and the overseas directors on recruitment and its effects on the health systems of the source countries. Difficulties in the retention of staff were highlighted by both groups of directors. Other findings included the language and cultural differences experienced by the overseas nurses.

**Conclusion:**

Recruitment of overseas nurses should not be left to the individual employer even in the presence of government guidelines. An international effort from all the involved parties is required to formulate a solution to this complex issue in order to protect both the health systems of individual countries and the nurse's right to migrate.

## Background

Although nurse migration is not a new phenomenon, the current rates of flow are unprecedented. It is difficult to accurately assess the true level of migration [1]. In many countries registration data is used as a source of statistics. In 2004, 1018 new nurses from outside the European Union registered in Ireland. Of these, 603 (59%) were from India, 267 (26%) from the Philippines, and 46 (5%) from South Africa [2].

Until recent years, Ireland had an oversupply of nurses [3]. However, the recent economic growth from the mid 1990s to the present day has resulted in the expansion of jobs for nurses in Ireland, so much so, that the number of jobs exceeded the domestic supply of employed nurses [4]. Ireland now actively recruits nurses from overseas. Initially, this trend was indicative of migration from 'high-income to high-income' country, although in 2000 migration from 'low and middle-income to high income' country became increasingly important. This change was due to the Irish government's international recruitment drive launched in 2000, following shortages of nursing labour as a result of under-investment in the Irish nursing workforce and the export of Irish nursing labour over previous decades [5]. In 1998, the Commission on Nursing found that there was a significant shortage of nurses [6]. The main reasons for this are the change in status of nursing students from that of employee to supernumerary status, resulting in a greater demand for registered nurses to cover duties that might have traditionally been undertaken by students. The high turnover rates historically associated with nursing is also contributing to the shortage of nurses. High-income countries also face an increased demand for nurses due to the ageing workforce caring for increasing numbers of elderly people [7], and young women who have been traditional recruits into the profession, having more attractive alternative career choices [8].

Historically, nurse migration was mostly opportunistic or based on individual motivation and contacts [7]. In recent years, one of the strongest "pull" factors is said to be the large scale planned international recruitment practices of high-income countries [9,10].

Guidelines on recruitment have had little impact on the management of international migration of overseas nurses [11]. As these guidelines are not mandatory, many countries have chosen not to follow them and therefore they only serve to highlight good employment practices when adhered to.

International organizations such as the World Health Organization [12], the International Council of Nurses [13] and the International Labour Office [11] have all described the driving factors of migration. Other studies have focused on the economic impact to the source [14] and host [15] countries. However there is a lack of information available on the experiences and perceptions of overseas nurses and those involved in the recruitment process. As nurse migration is increasing globally the effects of such migration on both source and destination countries will be examined. There has been relatively little primary research on the issue of international recruitment and migration of health workers [16]. Research in this area tends to focus on the underlying factors of migration and the economic impact to the source countries. Little is known about the opinions of those involved in the recruitment process. This paper explores the opinions and experiences of directors of nursing in both Ireland and the source countries, as well as the personal experiences and opinions of the overseas nurses themselves, currently working in Ireland. The aim of this paper is to establish the perceptions and opinions of those involved in the recruitment process on their role and the effects recruitment has on both source and destination countries.

## Methods

Qualitative approaches are associated with subjective descriptions of life experiences, in order to develop a greater understanding of the issues being considered. The intention of this study is not to build theory, but to describe a lived experience; therefore a phenomenological approach was used. The goal of phenomenological research is to describe the world as experienced by the participants in the study in order to discover the common meanings and underlying empirical variations of a given phenomenon [17]. The origins of this approach can be traced back to the German Philosophers Husserl (1859 – 1939) and Heidegger (1889 – 1976). Heidegger's Phenomenology which has an ontological base was considered to be more appropriate to this study than Husserlian Phenomenology, which has an epistemological base [18]. Heidegger's Phenomenology was chosen due to its ability to focus in depth, on human experiences as they are lived [19].

### Data collection

The participants were purposefully selected, using the research question as a guide. Nurse shortages were most pronounced in the three Dublin Academic Teaching Hospitals (DATHs) [20] therefore study participants were selected from the Irish Directors of nursing (IDNs) and the senior nurses from these hospitals. Overseas Directors of nursing (ODNs) were selected from the Philippines and South Africa as these countries were identified among the main sources of nurses to Ireland [21]. Numerous phone calls and emails were made to hospitals in India to select ODNs, but these were unsuccessful. For practicality, one DATH was chosen to obtain a sample of overseas nurses (ONs) i.e. nurses who had migrated from other countries to Ireland. Of the ten ONs, five were from India and five from the Philippines. The mean age of the seven females was 29 years and for the three males was 30 years. The nurses had been in Ireland for between one and seven years.

IDNs and senior nurses in Dublin were included if they were involved in the recruitment process of ONs to work in Ireland. ODNs were included if their hospital had turnover rates attributed to migration of their nurses to high-income countries. ONs were excluded if they had been in Ireland for less than one year.

Data were collected through in-depth interviews with the 12 DNs (Directors of Nursing) (six from Irish based teaching hospitals, three from South Africa and three from the Philippines), and 10 ONs. The interviews lasted between 30 and 60 minutes and were semi-structured as this acknowledged the methodological preference for "gentle guidance" rather than "firm control" [22]. The interviews took place at a venue suitable to the participants, however, telephone interviews were conducted with the ODNs. Before the interview started the participants received information about the aim of the study, data analysis and confidentiality. They were also reminded they could refuse to participate in this study without fear of recrimination and they could withdraw their consent at any stage during the interview. All interviews were audio taped and transcribed verbatim. Ethical approval was sought and obtained from the relevant research ethics committee. Written permission was obtained from the Director of Nursing to gain access to nursing staff in the DATH, and informed consent was obtained from all participants.

### Data analysis

A 'bottom up' approach to coding [23] was used. This involved reading each interview transcript several times, then analysing the content by trying to see past a quote at face value. Quotes appearing to contain similar content were given the same code and each code was further analysed to find true meanings within their text. Clusters of themes emerged from these codes. The credibility of the data obtained was established by 'member validation'. This involves taking the analysis of the responses back to the participants (or 'members') to enable them to check or comment upon the interpretation [24]. The research should also be reproducible, therefore a 'decision trail' of the research process was made clear. A second researcher found similar themes when analysing the transcripts, indicating good reliability. Confirmability requires the researcher to show the way in which interpretations have been arrived at in the study. In this study the findings are presented in a clear and logical way.

## Results

Five main themes were identified: migratory intentions, the effects of recruitment/migration, workforce diversity, alternatives to recruitment, and compensation.

### Migratory intentions

All ONs stated that their reason for joining nursing was that it afforded them the opportunity to work abroad. Many were encouraged by their families to become a nurse, even if it was not their preferred career choice. The ODNs expressed concern at being able to train good quality nurses from individuals lacking the desire to work as a nurse. One ODN from South Africa stated: "...they are not nurses in their hearts...how can we make them nurses?"

The IDNs also acknowledged that nurses traditionally joined the profession as they saw it as a 'passport to the world'. All participants expressed strong feelings on the nurse's right to migrate. Even the ODNs expressed a strong belief in the nurse's right to migrate despite the negative effects of this on their health system. They expressed a difficulty in balancing their beliefs in this right against their duties in maintaining a service, although they placed no blame on the nurses: "Why blame the nurse? She must go, my heart says so. I must give her my blessing, although I know we are not coping without her, I will not ask her to stay." (ODN from South Africa).

The migratory intentions of many overseas nurses are influenced by family factors and the ease at which their spouse can find work. Without suitable employment, living costs and house prices are too high and therefore the nurse will consider moving. Some nurses stated, quite ardently, that they intended to return home and nothing would change their mind: "...like the bird migrating, I will return home...of course...it is my home." (ON from India).

Irrespective of the influencing factors, none of the overseas nurses expressed an intension of returning to clinical nursing in their home country. The main reason was the difficulty of returning to the poorer working conditions in the hospitals.

### Effects of recruitment/migration

The IDNs, senior nurses and ONs all identified positive effects of recruitment and migration. There was general agreement that the effect would be of benefit to the ON, his or her family, and the economy of the source country. All the ONs stated the benefits of sending their salary home to support their family, and some mentioned the personal benefits of the experience, such as being able to travel and to engage in further studies. Although the ODNs acknowledged these benefits to the individual nurses, their concerns on the detrimental effect on nursing and the health systems in their own country were noticeable. The nurses that migrate from low and middle-income countries tend to be experienced and highly skilled. Ultimately it is the patient that suffers from the absence of skill. However, the quality of nurses in the future is of great concern, as this would have disastrous effects on the health system of the developing country: "I am left with only novice nurses...our experienced ones go...who will teach the novice nurse? Patient complaints are frequent because our nurses are not efficient." (ODN from the Philippines).

The nurses who remain in low and middle-income countries are faced with increased workloads and rising stress levels. This has lead to increased sick leave and absenteeism, further de-motivating the remaining staff. As an indication of the seriousness of the problem, one ODN from South Africa, in an attempt to ease the nurses' workload and stress, asked them to lower their standards so that they could complete more work in less time. The loss of skill is felt in all sectors of the health system as nurses migrate from private, public and rural areas. Frustration and a sense of helplessness or even hopelessness at the situation was apparent from the dialogue with the ODNs. IDNs did not identify this negative effect to the source country. This thinking is reflected by the ON, who does not recognize the negative effect of migration on their home country: "I think there is what you call a brain drain...a lot of nurses are leaving, but we train a lot, at this stage we are coping." (ON from the Philippines). This contrasts starkly with the view of an ODN from South Africa who stated "...we are at a level of desperation..."

### Workforce diversity

As patients in high-income countries are becoming more diverse, the IDNs welcome the migration of overseas nurses, believing it to benefit both nurses and patients: "There is a sharing of learning which ultimately is good for nurse and patient care." (IDN). The overseas nurses had contrasting views on working as a nurse in Ireland. For some they viewed their experience very positively: "...it is so kinda cosmopolitan like working with a lot of different nationalities. Different cultures can work together as one and bring their own unique culture into work...it's so positive." (ON, the Philippines). However, many overseas nurses experienced difficulties with this multiculturalism, and chose not to integrate into society: "We cook our own food, live in our own way...when I go outside I do not mingle much with Irish friends." (ON from India). This created a sense of isolation for some nurses. The main reason for not integrating into society appeared to be the desire to retain their culture as they found it difficult to adapt to the Irish culture (which they described as being very different from their own culture). Cultural differences also present many challenges to IDNs. They all noted that a "sense of responsibility" for their work was not embedded in the culture of the ONs. This was seen as a major obstacle to their integration into the workforce: "...it is a culture thing with the lack of responsibility that the overseas nurses take for accountability for their practice." (IDN).

ONs felt stressed due to their awareness of this difference in their practice. The IDNs also expressed concern that this lack of responsibility could lead to further problems. Despite making up a significant percentage of the nursing workforce, overseas nurses occupy very few senior positions, something that is frustrating for the IDNs: "It took so much coaxing to get one to apply for the post...and she was so qualified." (IDN).

Another factor of concern to the IDNs was the language difficulties of overseas nurses. Good communication is fundamental to the nurse patient relationship. One IDN stated "its creating difficulties for our patients". The ONs expressed great difficulties with language and communication on their arrival, with one nurse from India describing it as "mental torture" and saying "it is very difficult...I cannot even answer the phone".

### Alternatives to recruitment

It is a shared belief that the recruitment of overseas nurses will continue for the foreseeable future. All the DNs shared the belief that alternatives to recruitment must be considered a priority, although currently this is not happening at a national level. Recommendations from the DNs included exploring the reasons why there is a large dependency on overseas nurses and ways of ensuring sufficient production and retention of nurses. The issue of skillmix within nursing was identified by each DN as a way of reducing the need for overseas recruitment, and one they felt was not receiving warranted attention. Skillmix can be defined as the different combinations of staff required to provide patient care [25]. The determination of the DNs in retaining their nurses was strongly expressed during the interviews. Each outlined their achievements at the institutional level, for example making annual leave and working hours more nurse friendly, and securing greater funding for nurse education and study leave. ODNs also eagerly outlined their attempts to enhance retention, for example creating "nurse of the month" awards amongst many other initiatives. All the DNs agreed that institutional measures would only be effective if remuneration of the nurse is addressed. If nurses are to be retained they need to be rewarded financially.

One of the main challenges facing nursing, identified by all the directors, is its lack of value within the health care systems. Both IDNs and ODNs acknowledged their dual role in ensuring that nurses felt valued within nursing, but also that nursing received its rightful place within the health system. In contrast, newly recruited ONs identified the lack of value on nursing in their home countries, but reported a very positive experience in Ireland.

### Compensation

The ODNs had strong feelings on the issues of compensation. Some suggested they should be compensated for the loss of nursing manpower and skill. This could be done by increasing the capacity of nurse training schools, although the loss of skill already was acknowledged and therefore there was concern regarding who would teach the nurses. One ODN stated that action was already being taken to try to avoid this situation: "We are working towards the closure of 22 nurse training schools because of the poor quality." (ODN from the Philippines).

The ODNs all believed that compensation would be most effective if used to increase the salaries of those nurses who remain in the source countries, as these nurses are paying the price of others migrating. The IDNs felt compensation was not warranted as the nurses chose to migrate, and received equal opportunities to their Irish counterparts, which they felt was enough compensation. However, all the DNs were doubtful of the government's willingness to agree to provide compensation.

## Discussion

The Irish and overseas DNs had opposing views on recruitment. Recruitment of ONs was seen as a necessity by the IDNs. The ODNs felt the recruitment processes of western countries were fuelling current nurse migration. The views of the Directors will continue to differ so long as they experience very different outcomes from recruitment. However, all directors agreed on the financial benefits of migration. Other benefits identified by the ODNs were the travel and educational opportunities, which was why they supported the migration of nurses. The ODNs showed great concern regarding the 'brain drain' as it is known to have detrimental effects on the health systems in sources countries [26]. The WHO has warned that the loss of skilled health care workers is bringing health care systems to a "state of collapse" [27]. The ODNs identified the increased stress and work levels of the nurses who remained in their country. This is leading even more nurses to migrate [28,29].

The findings indicate that the nurses had no intention of returning to nursing in their home country, if they return home at all. Kingma (2005) [30] described the "myth of return" as completely untrue, as most nurse migrants settle in countries outside of their own. The thinking by the IDNs that knowledge gained here will be useful back at home appears to be unfounded.

The benefits of a diverse workforce were keenly identified by the IDNs and some ONs. The culture change for many overseas nurses was difficult to cope with. Many people can easily identify their own culture by virtue of being immersed in another, and may be keen to maintain their identities and cultural values [31]. IDNs reported that the ONs were reluctant to take responsibility in the workplace and go forward for senior positions, despite many of them being more than able for the job. One possible reason for this was highlighted in a study conducted by McAuliffe *et al*. (2002) [32] with 81 ONs recruited to Ireland. When compared to their Irish counterparts, it was apparent that overseas nurses were experiencing difficulties assuming the autonomy and control over their own professional nursing practice that is common in Irish nursing practice. These included issues such as care planning and making nursing care decisions for patients in a system that required a greater degree of subjectivity. The findings highlighted that respondents were more familiar with a system in which nursing care was delivered with "objective planned interventions" and where interventions were guided by adherence to policies and procedures. These findings are consistent with those of Daniel et al. [33] and Charest [34], who found that ONs work was directed by endorsements from doctors and their role was comparable to that of the doctor's assistant. The emphasis for nurses was on carrying out the legitimate orders of the physician and that nurse autonomy and being in control of their practice in terms of exercising their own clinical judgment was not present in their professional values. Martin et al. [35] have also reported a seemingly more paternalistic relationship with physicians. It would be interesting to explore whether this phenomenon occurs in other countries that nurses migrate to. Another reason may be that overseas nurses have unequal career opportunities. An in-depth study by Obrey et al. [36] found that overseas black and minority ethnic nurses working in England felt they had unequal opportunities for career advancement.

Language was identified by the IDNs as being a major influencing factor in their decision on where to recruit from. The nurses' training must have been conducted in English and nurses must have passed an international English test before a position of employment can be offered. Although the Directors recognized a problem with language, they did so only from the patient's perspective. It is essential that the nurses feel supported through this difficulty as it may negatively effect their decision to stay.

Alternatives to recruitment were identified by the IDNs, although until Ireland supplies enough nurses to meet its demand, recruitment will continue. This is true for all Western countries currently experiencing nurse shortages [37]. Underproduction has been a significant causative factor of current shortages globally [11]. Skillmix was seen as a solution to reduce the need for ONs, although both Irish and overseas DNs encountered difficulties with its introduction, with the absence of National leadership on this issue being a main contributor. IDNs stressed the importance of maintaining a quality learning environment if student numbers are to be increased. In maintaining a quality environment here, there is continued reliance on ONs, thus fuelling migration. As well as affecting the quality of the learning environment in source countries, the quality of the health service in its totality would be affected. This was not identified by the IDNs. All Directors identified the lack of value within the health system for nursing. ONs however, only identified a lack of value in nursing in their home countries, and not in Ireland. This may present problems with one cohort of nurses feeling undervalued while another feels the opposite. The ICN warned of the damaging consequences of placing new recruits into a dysfunctional system [38].

Although the ODNs felt compensation for the loss of their nurses was a very justified request, it is not a straightforward solution. Compensation is one of the key recommendations of the Commonwealth Code on overseas recruitment. However, many countries have refused to sign up to the code because of the compensation clause.

### Limitations

Although phenomenological studies generally have a small number of participants, including more ODNs from a wider range of countries may have added breadth to some of the themes. In particular, as India is a major source country for recruitment of nurses to Ireland, the inclusion of ODNs from India would have strengthened the findings of the study. ONs that were recruited to Ireland, but have since left were not included in this study. Inclusion of these nurses would have helped provide knowledge of factors influencing their decision to migrate further or return home.

### Practical implications

Proactive effective steps are vital to protect the health systems of source countries. Improvements in working conditions for nurses in their source countries are needed, as this study identified increasing stress and work levels of the nurses. The findings also indicated that the poor working conditions for nurses in their source countries is likely to contribute to their lack of desire to return home. Greater encouragement and support should be provided to overseas nurses to progress in their career. This could be provided through improved communication to overseas nurses regarding training and career opportunities, and providing an on-going support system for nurses through regular meetings or discussion groups with management to discuss progress and other relevant issues. There is a need for further investigation to identify whether barriers exist in the workplace preventing ONs seeking promotion. Furthermore, the issue of skillmix in nursing should be addressed through the creation of a national task force involving all relevant stakeholders. Language barriers were identified as an issue for integration of overseas nurses as well as prerequisites of quality nursing care. A review of the International English test and its suitability in assessing proficiency in English speaking for nurses should be conducted. The Irish government should consider establishing language courses for migrants.

## Conclusion

The recruitment and migration of nurses is set to continue for the foreseeable future. It has brought many benefits to the Irish healthcare system, the ON and their families. However, if it continues at the current pace, the healthcare system of the source countries will be severely damaged. Action is needed at an international level in order to protect the health systems of source countries currently relied on by billions of people. Countries no longer have any ownership of the healthcare professionals they train. In our globalised world, nurses have become global public goods. It is no longer possible for one country to solve the migration problem. Even bilateral agreements between countries provide limited control over migration flows. A solution to this complex problem must involve *all *relevant stakeholders and a commitment to ensure that the nurse's right to migrate is preserved while protecting the collective healthcare needs of the involved population. Unless this is done, wealthy countries will continue to have their health systems supported by those countries whose systems are close to collapse.

## Competing interests

The author(s) declare that they have no competing interests.

## Authors' contributions

PHT and EMcA participated in the design and analysis of the study. PHT conducted the research. All authors contributed to the interpretation of the data. LAW drafted the paper. All authors contributed to the final manuscript.
